# Three-Dimensional Ankle Exercise with Combined Isotonic Technique for an Obese Subject with Plantar Fasciitis: A Case Study

**DOI:** 10.3390/medicina56040190

**Published:** 2020-04-21

**Authors:** Kyung-Sun Lee, Du-Jin Park

**Affiliations:** 1 Department of Industrial Health, College of Health Sciences, Catholic University of Pusan, Busan 46252, Korea; ksunlee@cup.ac.kr; 2 Department of Physical Therapy, College of Health Sciences, Catholic University of Pusan, Busan 46252, Korea

**Keywords:** ankle exercise, isotonic technique, obesity, plantar fasciitis, foot function

## Abstract

*Background and objectives:* Obese people have many foot-related disorders and plantar fasciitis (PF) is the most common disorder among them. However, research on the role of therapeutic exercises in PF is lacking and there is no evidence to suggest its benefits. As such, a further insight into therapeutic exercises is needed within this group. This case study investigated the effect of three-dimensional (3D) ankle exercises using a combined isotonic (CI) technique on function and balance in an obese subject with PF. *Material and methods:* The subject in this study was a 28-year-old obese woman who was diagnosed with PF by an orthopedic surgeon. A 3D ankle exercise program was commenced three times a week for 15 min over 4 weeks. The evaluations were conducted at five intervals: pre-test, and at 1, 2, 3 and 4 weeks from the initiation of the intervention. The tests were conducted in the following order: the patient-specific functional scale test (PSFS), an ultrasound of the plantar fascia, the heel pressure and balance test, the pressure pain threshold (PPT), and the 4-way ankle strength test. *Results:* The mean score of the PSFS test reduced by 70.55% after 4 weeks of the intervention. The thickness of the plantar fascia and heel pressure measured during single-leg standing decreased by 6.67% and 10.37%, respectively, after 4 weeks of the intervention. The anteroposterior and medial-lateral balance ability showed improvements of 8.29% and 8.61%, respectively, after 4 weeks of the intervention. The PPT improved by 38.01% after 4 weeks of the intervention. In the 4-way ankle strength test, dorsiflexion, plantar flexion, inversion, and eversion increased by 14.46%, 9.63%, 4.3% and 13.25%, respectively, after 4 weeks of the intervention. *Conclusion:* 3D ankle exercises utilizing the CI technique were shown to be effective in improving foot function, pressure pain, and muscle strength in dorsiflexion and inversion in an obese patient with PF.

## 1. Introduction

In the United States, it is estimated that approximately 10% of the total population will experience plantar fasciitis (PF) or plantar heel pain [1]. An elevated body mass index (BMI) is the only factor associated with PF [2]. A recent retrospective study found a high prevalence of obesity and female gender among patients with PF and tibialis posterior tendonitis [3]. Obesity is classified based on BMI and, for Asians, those with a BMI of 25 or higher are categorized as obese [4]. Foot pain is more likely to develop in obese people compared to those of a normal weight [5,6], and when walking, they are more likely to develop pes planus or show a high plantar pressure [7]. Recent studies have addressed the risk of developing PF as well as foot dysfunction in obese individuals, drawing attention to the need for a preventive intervention [6,8]. In addition, PF has been reported to be associated with a reduced medial longitudinal arch height of the foot, decreased muscle strength of ankle eversion, and a decline in balance ability compared to those with normal weight [9].

Simons et al. have suggested that trigger points in the gastrocnemius muscles may be involved in the development of plantar heel pain [10]. The authors of a previous study reported that muscle stiffness at the site of a trigger point was 50% greater than that of the surrounding muscle tissue [11]. Additionally, it has been reported that a stretching program including the triceps surae and the plantar fascia is moderately effective in the treatment of PF [12]. McKeon et al. (2015) argued that the harmonious activity of the intrinsic and extrinsic muscles is pivotal for foot stability [13]. A previous study found that an intervention combined with eccentric and concentric training improved ankle strength and proprioception in athletes with functional ankle instability [14]. A combined isotonic (CI) technique in proprioceptive neuromuscular facilitation (PNF) enables the concentric, eccentric, and stabilizing contractions of agonists without relaxation in order to improve coordination, joint range of motion, muscle strength, and eccentric control [15]. Therefore, this case study aimed to investigate the effect of three-dimensional (3D) ankle exercises using the combined isotonic (CI) technique on function and balance in an obese subject with PF.

## 2. Materials and Methods

### 2.1. Subject

The subject in this study was a 28-year-old woman and college student. She had a constant foot pain due to increased body weight and visited a local hospital. The diagnosis of PF was based on patient history and the results of the radiography, ultrasonography, and physical examination conducted by an orthopedic surgeon. The patient had no foot-related past history, and special hobbies or leisure activities. She received the modality treatment once, including ultrasound and electrotherapy, on the day she was diagnosed with PF. Since then, she has not received and/or experienced any treatment or ankle therapeutic exercises. The subject’s height, weight, and BMI were 158 cm, 70 kg and 28.04 kg/m^2^, respectively. The subject did not have any musculoskeletal, neurological, or other diseases except PF, and no heel spurs were observed on the radiographs. However, the patient experienced discomfort and pain in the dominant right sole while walking that had persisted for 6 months. The dominant leg was defined as the one that kicks a ball farther [16]. Through an interview, the participant was informed about the 4-week exercise program and it was explained that the entire course of the study would be conducted in compliance with the Declaration of Helsinki. The study was approved by the Catholic University of Pusan Institutional Review Board (CUPIRB-2019-060).

### 2.2. Measurement Methods and Tools

#### 2.2.1. Pressure Pain Threshold (PPT)

One typical symptom of subjects with PF is pain in the heel area. An algometer (JTECH Medical; Midvale, UT, USA) was placed vertically at the painful points on the medial heel where the patient experienced the strongest pain [17,18]. The PPT describes the amount of pressure needed for the first sensation of pain. The subject was asked to say “ah” when her sensation changed from pressure to pain. Then, the application of pressure was stopped, and the pressure pain measurements were recorded. The measurement was performed three times in total and a 30-s rest was provided between measurements.

#### 2.2.2. Questionnaire for Measuring Foot Function

A patient-specific functional scale (PSFS) was used to measure the foot function of the subject. The PSFS is a tool designed for easy use while maintaining validity and reliability in assessing musculoskeletal function in various patients in clinical practices [19]. Patients are assessed for three to five major activities that they cannot perform or have difficulties performing, with a score of 0 for incapability and 10 for performance at the level before injury [20]. This study measured the levels of foot function during three major activities through an interview with the subject [17]. Of the three major activities, the first item (PSFS 1) was “activity after getting up” (as it was shown that foot pain was often most intense with the first steps after waking up) [21], the second item (PSFS 2) was “jogging”, and the third item (PSFS 3) was “hill climbing”.

#### 2.2.3. 4-Way Ankle Strength Test

To measure the strength of the ankle joint, dorsiflexion, plantar flexion, inversion, and eversion were performed, and muscle strength was evaluated based on the reference posture presented by Hislop et al. (2013) [22]. The ankle strength test was performed using a hand-held dynamometer (Commander Muscle Tester, JTECH Medical). To normalize values of ankle muscle strength, muscle strength (N) displayed on the dynamometer was divided by the weight of the subject [8,9,23].

#### 2.2.4. Heel Pressure and Balance Test

The Tekscan pressure mapping tool (Tekscan; South Boston, MA, USA) was used to measure the change in heel pressure and center of pressure (COP) while the subject stood on one leg. Single-leg standing was performed for 5 s, and changes in pressure and COP for 3 s excluding the first and last second were used in this study. Changes in heel pressure and COP were measured three times in total.

#### 2.2.5. Measurement of Thickness of Plantar Fascia

**Figure 1:**
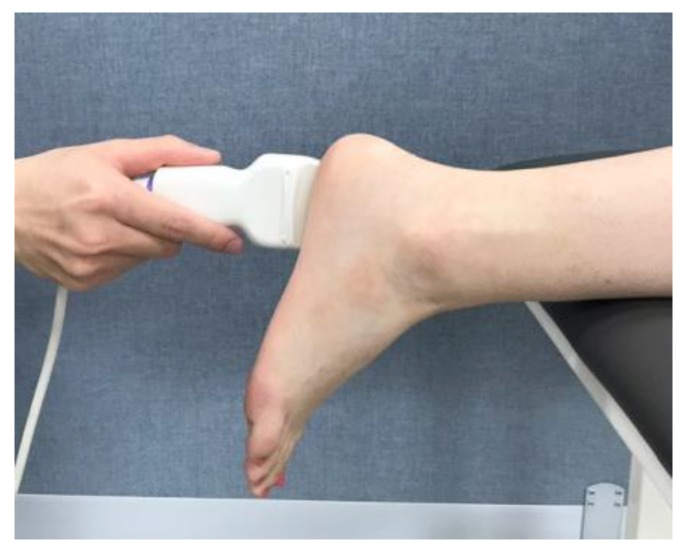
Ultrasonography of plantar fascia.

To compare the change in the thickness of the plantar fascia before and after the intervention, an ultrasonography (Prosound 2, Hitachi Aloka Medical; Tokyo, Japan) was performed. The participant was positioned in prone with the right foot over the edge of the table and the ankle in neutral [24]. Ultrasonic images and captures were measured in B-mode and static condition. The transducer with a 6–13 MHz linear probe and 40 mm scanning width (UST-5551) was placed over the plantar surface of the heel approximately 0.5 cm medial to the midline longitudinal axis of the foot (Figure 1) [24]. This study used intra-class correlation coefficients (ICC) to investigate the intra-rater reliability of the measurement of the thickness of the plantar fascia. ICC_(3,1)_ showed 0.86 (0.55–0.98, 95% confidence interval).

### 2.3. Intervention

**Figure 2:**
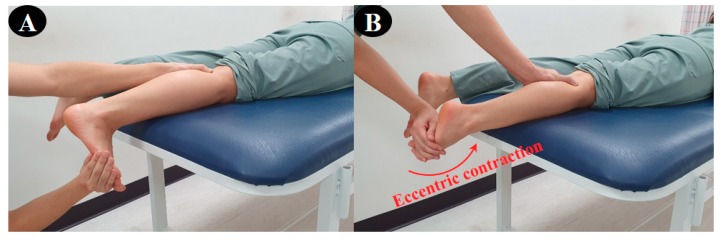
3D ankle extension exercise: (**A**) start position, (**B**) end position.

**Figure 3:**
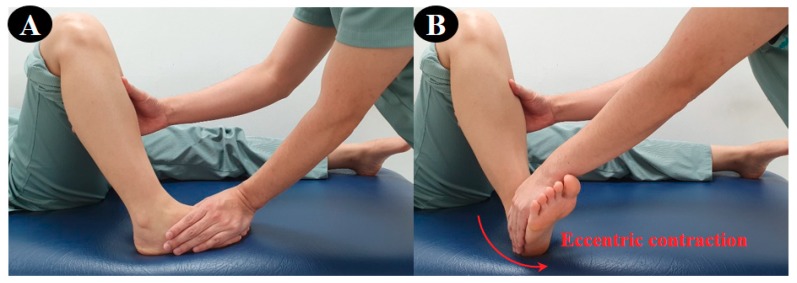
3D ankle flexion exercise: (**A**) start position, (**B**) end position.

For the 3D exercise interventions in this study, the flexion-abduction-internal rotation and extension-adduction-external rotation, which are the two diagonal (D2) patterns from the PNF leg patterns, were used. The CI technique was applied stressing only the ankle movement in the D2 flexion–extension pattern. The CI technique is a representative method among agonist techniques for PNF, which combines concentric, eccentric, and stabilizing contractions to provide functional training on coordination, joint range of motion, muscle strength and the eccentric control of the subject [15]. First, the participant performed the D2 extension pattern (plantar–flexion–supination–inversion–toe flexion) using the CI technique in the prone position according to the resistance of the therapist. (Figure 2). Second, she performed the D2 flexion pattern (dorsi–flexion–pronation–eversion–toe extension) using the CI technique, and the counter pattern in the crook lying position (Figure 3). In Figure 1 and Figure 2, the red arrow indicates the direction of application of eccentric contraction during the 3D foot-ankle exercises.

**Figure 4:**
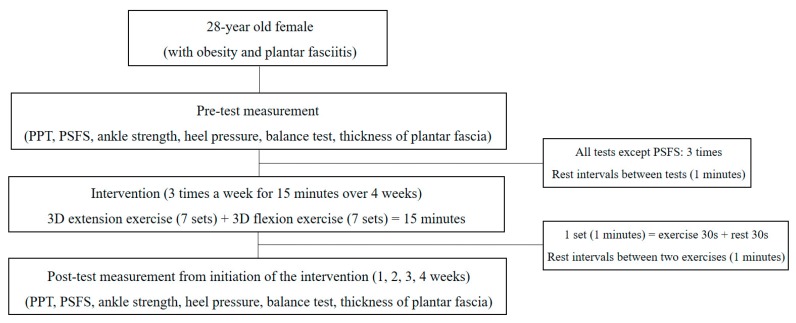
Study flowchart.

Training for the 3D ankle exercises was conducted for about 15 min before the intervention following which, the intervention was performed (Figure 4). The intervention was carried out three times a week for 15 min over 4 weeks. The intervention procedure was as follows: 15 s of the CI technique was applied during flexion and extension exercises followed by 30 s of the intervention. In order to prevent muscle fatigue, a rest period of 30 s was included to bring the set to a total of 1 min. In addition, efforts were made to minimize muscle fatigue by providing a 1-min break before conducting the counter pattern. The configuration of the set was applied in the same way, and a total of 14 sets (seven sets for each pattern) were performed for 15 min.

### 2.4. Experimental Procedure

The evaluations were conducted at five intervals: pre-test, and at 1, 2, 3 and 4 weeks from initiation of the intervention. Except for the PSFS, the other tests were performed three times in total, and the mean value of the three measurements was used in this study. In order to minimize the interaction between tests, the tests were conducted in the following order: PSFS, an ultrasound of the plantar fascia, the heel pressure and balance test, the PPT, and the 4-way ankle strength test.

## 3. Results

**Figure 5:**
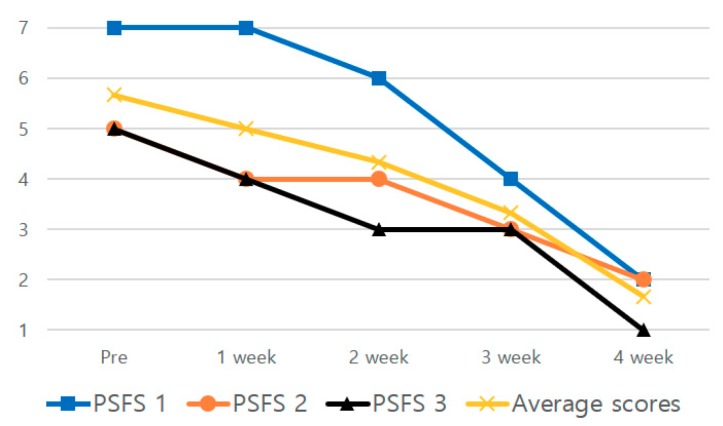
Changes in the patient-specific function scale over 4 weeks.

**Figure 6:**
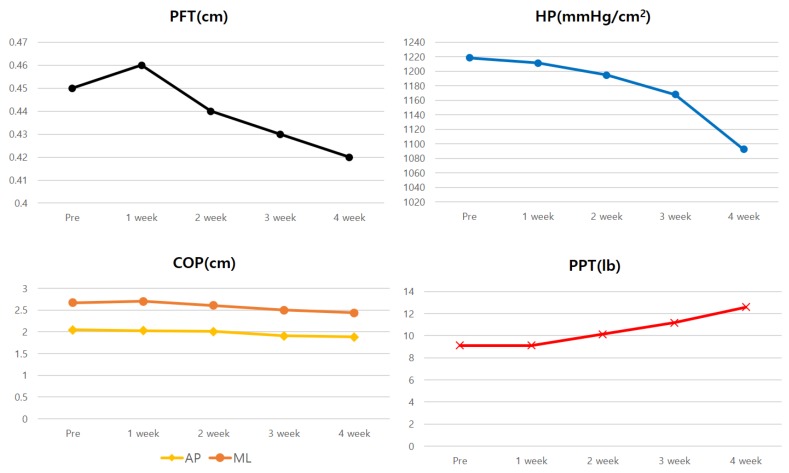
Changes in plantar fascia thickness (PFT), heel pressure (HP), center of pressure (COP), pressure pain threshold (PPT) over 4 weeks.

**Figure 7:**
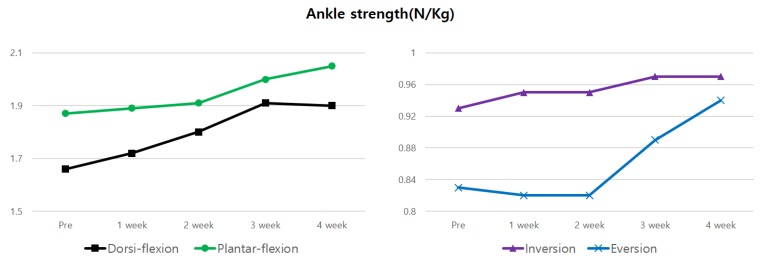
Changes in 4-way ankle strength over 4 weeks.

The mean score of the PSFS reduced by 70.55% after 4 weeks of the intervention compared to the scores before the intervention (Table 1, Figure 5). The thickness of the plantar fascia and heel pressure during single-leg standing decreased by 6.67% and 10.37%, respectively, after 4 weeks of the intervention, compared to the results before the intervention (Table 2, Figure 6). The anteroposterior and medial-lateral balance ability showed an improvement of 8.29% and 8.61%, respectively, after 4 weeks of the intervention compared to those before the intervention (Table 2, Figure 6). The pressure pain improved by 38.01% after 4 weeks of the intervention (Table 2, Figure 6). In the 4-way ankle strength test, dorsiflexion, plantar flexion, inversion, and eversion increased by 14.46%, 9.63%, 4.3% and 13.25%, respectively, after 4 weeks of the intervention (Table 3, Figure 7).

## 4. Discussion

A very important factor in the diagnosis of PF is the presence of pressure pain in the heel of the foot [25]. In this study, pressure pain reduced by 38% after 4 weeks of the intervention, thereby indicating a positive effect of 3D ankle exercises. Popular and traditional treatments for PF include stretching exercises for the plantar fascia as well as strengthening exercises for the intrinsic muscles of the foot [26,27]. In the 3D ankle exercises, the flexion exercise was performed by inducing maximum dorsiflexion while supporting the heel, which led to an improvement in the Achilles tendon and triceps surae stiffness, and increased the muscle’s flexibility [28]. Toe flexion and adduction are induced during the 3D extension exercise, which increases the activity of the intrinsic muscles of the foot [29]. As a result, it is thought that the strengthening of the intrinsic muscles of the foot will reduce the load on the plantar fascia, a passive structure, while maintaining the height of the medial longitudinal arch of the foot, resulting in a decrease in pressure pain. Furthermore, this led to a decrease in heel pressure during single-leg standing, resulting in a significant improvement in pain and discomfort of 70% after 4 weeks, compared to the values before the intervention that were measured while performing the major functional activities of the PSFS.

An important predictor affecting ankle balance is ankle muscle strength [30]. In particular, it has been reported that obese subjects have a higher risk of ankle sprain due to decreased ankle muscle strength for eversion compared to those with a normal weight [9]. Ankle sprains are caused by forces that cause excessive inversion and plantar flexion, and eccentric training on dorsiflexion and eversion is needed to prevent this [31]. In this study, 3D ankle exercises showed an increase in muscle strength of more than 13% for dorsiflexion and eversion. This increase in muscle strength results from eccentric contraction training, and eccentric contraction for these muscles can improve joint position sense, which is a type of proprioception [14]. In addition, concentric and eccentric contraction for dorsiflexion may lead to a better loading response at initial contact during the walking cycle. This is thought to have a positive effect on the results of the PSFS.

In obese subjects, in particular, the height of the medial longitudinal arch of the foot decreases, leading to a reduction in anteroposterior balance ability compared to those with a normal weight during single-leg standing [9]. In the results of this study, the anteroposterior and medial–lateral balance abilities improved by 8% after 4 weeks of the intervention compared to those before the intervention. This is thought to be a result of enhancing coordination as well as strengthening the intrinsic and extrinsic muscles of the foot through the CI technique [15].

A limitation of this study was that the long-term effects could not be examined after the completion of the intervention, and that due to the lack of information on the medial longitudinal arch height of the foot, the change in the height of the medial longitudinal arch could not be observed in detail. Musculoskeletal ultrasounds have been widely used in assessing musculoskeletal disorders because of the advantages of lower cost and portability [32,33]. Based on this, the present study measured the thickness of the plantar fascia, but did not observe the stiffness of the calf and plantar fascia, as well as the cross-section area of the intrinsic muscles. In order to identify the effect of intervention for plantar fascia, it is necessary to compare the thicknesses of both plantar fascia using ultrasonography.

## 5. Conclusions

3D ankle exercises with the CI technique were shown to be effective in improving foot function, pressure pain, and muscle strength in dorsiflexion and inversion in an obese subject with PF. We envision that the benefits of therapeutic exercises will be recognized for their ability to improve functional activity and foot pain in people with PF, and as such they will be implemented in the treatment of PF.

## Figures and Tables

**Table 1:** Outcomes of the PSFS scores.

	Pre	1 Weeks	2 Weeks	3 Weeks	4 Weeks
PSFS ^1^ 1	7	7	6	4	2
PSFS ^1^ 2	5	4	4	3	2
PSFS ^1^ 3	5	4	3	3	1
Average scores	5.67	5	4.33	3.33	1.67

^1^ PSFS: patient-specific function scale.

**Table 2:** Outcomes of thickness of plantar fascia, heel pressure, balance ability and PPT.

	Pre	1 Weeks	2 Weeks	3 Weeks	4 Weeks
PFT ^1^ (cm)	0.45	0.46	0.44	0.43	0.42
HP ^2^ (mmHg/cm^2^)	1218.67	1211.33	1194.67	1168.00	1092.33
AP ^3^-COP ^4^ (cm)	2.05	2.03	2.01	1.91	1.88
ML ^5^-COP ^4^ (cm)	2.67	2.70	2.61	2.50	2.44
PPT ^6^ (lb)	9.13	9.13	10.13	11.17	12.6

^1^ PFT: plantar fascia thickness, ^2^ HP: heel pressure, ^3^ AP: anterior posterior, ^4^ COP: center of pressure, ^5^ ML: medial lateral, ^6^ PPT: pressure pain threshold.

**Table 3:** Outcomes of 4-way ankle strength.

	Pre	1 Weeks	2 Weeks	3 Weeks	4 Weeks
Dorsi-flexion (N/Kg)	1.66	1.72	1.80	1.91	1.90
Plantar-flexion (N/Kg)	1.87	1.89	1.91	2.00	2.05
Inversion (N/Kg)	0.93	0.95	0.95	0.97	0.97
Eversion (N/Kg)	0.83	0.82	0.82	0.89	0.94
